# Gradient-Boosting Algorithm for Microwave Breast Lesion Classification—SAFE Clinical Investigation

**DOI:** 10.3390/diagnostics12123151

**Published:** 2022-12-13

**Authors:** Aleksandar Janjic, Ibrahim Akduman, Mehmet Cayoren, Onur Bugdayci, Mustafa Erkin Aribal

**Affiliations:** 1 Mitos Medical Technologies, ITU Ayazaga Ari Teknokent 2-B Block 2-2-E, 34469 Istanbul, Turkey; 2 Electrical and Electronics Engineering Faculty, Istanbul Technical University, 34469 Istanbul, Turkey; 3 Department of Radiology, School of Medicine, Marmara University, 34899 Istanbul, Turkey; 4 Radiology Department, Breast Health Center, Altunizade Hospital, Acibadem M.A.A. University, 34684 Istanbul, Turkey

**Keywords:** microwave breast imaging (MBI), SAFE, breast lesion classification, machine learning

## Abstract

(1) Background: Microwave breast imaging (MBI) is a promising breast-imaging technology that uses harmless electromagnetic waves to radiate the breast and assess its internal structure. It utilizes the difference in dielectric properties of healthy and cancerous tissue, as well as the dielectric difference between different cancerous tissue types to identify anomalies inside the breast and make further clinical predictions. In this study, we evaluate the capability of our upgraded MBI device to provide breast tissue pathology. (2) Methods: Only patients who were due to undergo biopsy were included in the study. A machine learning (ML) approach, namely Gradient Boosting, was used to understand information from the frequency spectrum, collected via SAFE, and provide breast tissue pathology. (3) Results: A total of 54 patients were involved in the study: 29 of them had benign and 25 had malignant findings. SAFE acquired 20 true-positive, 24 true-negative, 4 false-positive and 4 false-negative findings, achieving the sensitivity, specificity and accuracy of 80%, 83% and 81%, respectively. (4) Conclusions: The use of harmless tissue radiation indicates that SAFE can be used to provide the breast pathology of women of any age without safety restrictions. Results indicate that SAFE is capable of providing breast pathology at a high rate, encouraging further clinical investigations.

## 1. Introduction

Breast cancer is the most diagnosed cancer in women, accounting for 11.7% of all cancer diagnoses. It is the leading cause of cancer-related deaths among women [1]. Breast cancer death rates decreased over the years, which is believed to be the result of more regular early screenings and better treatments. The high 5-year survival rates are reported for stage 1 and 2 (99%) and stage 3 (86%) diagnosed cancers, contrary to the low rate of 26% for those diagnosed with stage 4 breast cancer [2]. Traditional diagnostic techniques, such as mammography, have been widely used for breast cancer screening and diagnostics, with proven positive effects on the breast-related cancer death rate [3]. Still, mammography is limited by several risk factors such as false negativity in dense breasts, exposure to ionizing radiation, false-positive examinations and patient discomfort [4,5,6,7,8,9,10,11,12,13]. It is also discouraged in women under the age of 40, due to the increased breast density in younger patients, where sensitivity reported varies between 62% and 69% [11]. Ultrasonography and magnetic resonance imaging (MRI) are additional techniques that can be used for breast cancer screening and diagnostics. Ultrasonography can be operator-dependent and can increase the number of benign biopsies, leading to patient anxiety and higher hospital costs [14,15,16]. MRI is mostly recommended for high-risk patient groups and is not considered as practical for early screening and diagnostics due to higher hospital costs [12,17,18,19].

Microwave breast imaging (MBI) is a novel imaging technique that has the potential to greatly contribute to the field of breast cancer early screening and diagnostics due to its non-invasive and non-ionizing nature. MBI utilizes the difference in dielectric properties of healthy and cancerous breast tissue, as well as the difference in dielectric properties of different cancerous tissue types, to detect the presence of anomalies inside the breast and provide their pathology. The difference in dielectric properties is confirmed and reported by various studies [20,21,22,23,24,25,26,27]. The non-ionizing nature of MBI allows safe and repeated examinations of all women, regardless of their age, which can especially be beneficial for women at high risk of developing breast cancer, where there is a necessity to start screenings at an early age.

A wide range of clinical studies on MBI have been conducted and published up to this point, with a comprehensive overview given by (Benny et al., 2020; Kwon and Lee, 2016; O’Loughlin et al., 2018) [28,29,30]. At the time of writing this, three prototypes have undergone clinical trials with a significant number of patients. The University of Bristol and Micrima, UK, have conducted the largest scale studies, with 225 patients involved. The reported sensitivity of their MBI system, namely MARIA, was 76% [31,32]. Beside Micrima, Umbria Bioengineering Technologies (UBT), Italy, clinically tested their device, MammoWave, on 58 patients reporting sensitivity of 74% [33]. Recently, UBT published its work considering automated breast lesion identification. A machine learning (ML) model, namely the support vector machine (SVM), was implemented on the raw data of 35 patients, achieving accuracy, sensitivity, and specificity of 91%, 84.4% and 97.2%, respectively [34]. Mitos Medical Technologies (MITOS), Turkey, in collaboration with Istanbul Technical University, Turkey, published the results of their clinical research study conducted with their SAFE (Scan and Find Early) MBI device, where the reported sensitivity was 63% [35]. Recently, MITOS analyzed the contribution of adaptive boosting (AdaBoost) of the random forest algorithm to automated breast lesion classification. The dataset from the previous study was used. The reported sensitivity, specificity and accuracy of SAFE in this study were 79%, 77% and 78%, respectively. This work is still under review [36]. Besides the mentioned prototypes, the Microwave Vision (MVG) Medical Imaging department, France, developed the MBI device named Wavelia and tested it in a small-scale study of 24 patients. They reported that they successfully separated benign from malignant lesions in 88.5% of the cases, whereas their malignancy risk calculation was deemed useful in 70.6% of the cases [37,38,39,40].

Building on our previous research [35,36], the aim of this study is to use a new dataset containing 54 patients and test the new machine learning (ML) approach, namely Gradient Boosting, to compare new study findings with the previous ones, and find the best-fitting ML model for automated breast lesion classification. The procedure, as in our previous study, is verified by the pathological results provided through the biopsy (used as the gold standard in the study).

## 2. Materials and Methods

The study was approved by the Ethics committee of Marmara University School of Medicine (Protocol number: 70737436-050.06.04; Date of approval: 9 June 2014). Patient participation was voluntary and written informed consent was obtained from all participants. All protocols and procedures were in accordance with both institutional and national ethical standards in research and with the World Medical Association Declaration of Helsinki.

Only the patients considered for the biopsy were included in the study. The SAFE scan is performed prior to the biopsy due to the post-biopsy impact mentioned by Fasoula et al. [37]. Lesion pathology was provided after the biopsy was performed. All patients were biopsied using a 14G disposable automatic True-Cut needle under ultrasound guidance. Histopathological verification was used as the gold standard. Patients were divided into subgroups according to areas of interest, such as age, density and breast size. Breast density was acquired for the patients who underwent mammography or MRI examinations prior to or after the biopsy. Density information was unknown for the patients that only underwent ultrasound. Breast density was evaluated based on the BI-RADS, according to the following scale: A—almost entirely fat; B—scattered areas of fibroglandular tissue; C—heterogeneously dense; D—extremely dense [41]. As the number of samples were limited, we divided patients into two density groups: non-dense (BI-RADS A and B) and dense (BI-RADS C and D). Patients were divided into two age groups: young, with the ages 18–39 years, and older, with the ages 40 years and above. The age boundary was decided based on the recommended age to start routine mammography examinations (aged 40 years and above). Breast size was decided based on the breast cups used to hold the breast during the scan. Four breast cups were made available where, for each scan, the choice of the cup was subjective and was made by the medical technician performing the scan.

**Figure 1:**
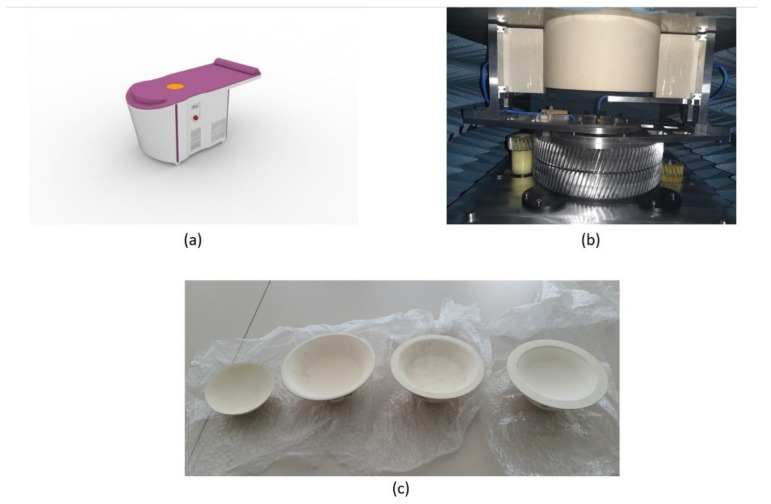
SAFE (Scan and Find Early) microwave breast cancer imaging device: (**a**) SAFE’s new portable device design; (**b**) scanning unit consisting of one transmitting and one receiving antenna rotating around breast holder; (**c**) breast cups intended to match different breast sizes.

The scanning unit consisted of one transmitting (*T_x_*) and one receiving (*R_x_*) antenna, which rotated around the azimuth to collect the backscattered signals from different angular positions. The *T_x_* and *R_x_* were connected to the 2-port Vector Network Analyzer (VNA), which, compared to our previous study [35], was changed from the Agilent N5230A PNA-L to the P9372A Keysight USB VNA model [42]. One of the reasons for the change of VNA was its size, which directly correlates with the portability of the MBI device. P9372A Keysight USB VNA is much smaller in size, allowing us to make a more compact device design. The frequency band used in [35,36] ranged from 1 to 8 GHz, while in this study, the frequency range was from 0.6 to 8 GHz. The same step of 200 MHz was applied, leading to the 38 frequency samples (*NF*). The backscattered signals were collected in a bistatic fashion, where for each *T_x_* position there were a number of *R_x_* positions. In the current setup, the number of *T_x_* as well as *R_x_* positions (*NT_x_* and *NR_x_*) were 36, meaning that both antennas have a displacement of *10^o^*. For each breast, raw data in the form of complex matrix *S*_21_, with the size of 36 × 36 × 38 (*NT_x_, NR_x_, NF*), were acquired. The hub, in which the scanning unit was placed, was completely covered with absorbers in order to diminish the noise that can arise from mechanical movement. The top of the hub was equipped with the hole in which the breast cup was placed. During the examination, the patient was required to lie prone on the table with one breast inserted into the cup. The cup is placed not just as a breast holder, but also as an impedance-matching medium. In Figure 1, the device design, and those of the scanning unit and breast cups, are presented. The scanning process was performed by the medical staff of Marmara University, School of Medicine radiological department. Reported data-acquisition time was approximately 10 min per patient scan, while in our previous study [35], this reported time was approximately 20 min. The significant decrease in scanning time provides the opportunity for more scans per examination day.

In order to make an automated breast lesion classification, *S*_21_ parameters (parameters proportional to the electromagnetic field emerging after the incident wave, radiated by transmitting antenna, interact with the breast) from each breast cancer were collected and used. To classify backscattered signals as benign or malignant, we employed only real parts of *S*_21_ as features for signal classification. The two-sample t-test was conducted to evaluate whether the real *S*_21_ could be used to classify the backscattered signals of benign and malignant breast tissue. The null hypothesis of the test (*H*_0_) assumed that the two sets being tested were not statistically significantly different, and thus were unsuitable to be used for classification purposes. This indicates that the alternative hypothesis (*H*_1_), showing the existence of significant statistical difference, should be accepted. The null hypothesis is rejected if the p-value is less than the confidence level chosen (*p < α*). The confidence level chosen in this study was 0.05. A confidence level of 1.44 × 10^−7^ was achieved, indicating that the alternative hypothesis was valid and that real *S*_21_ can be used for automated breast lesion classification. 

The Gradient-Boosting classifier was used to identify the backscattered signals from the breast. The algorithm was trained based on pathological results (benign or malignant). Gradient Boosting is an ensemble method where several weak learners are sequentially combined into a strong learner. At each iteration, the new model is minimizing the loss function of its predecessor, until the optimal estimate of the target variable is achieved. The model does not modify the sample distribution. This means that the weak learner is not trained on the newly sampled distribution but is rather trained on the remaining errors of the strong learner. At each iteration, the weak learner is fitted to newly computed pseudo-residuals, where the contribution of the weak learner is computed based on the gradient descendent optimization process, rather than by its performance on the newly distributed sample. The overall error of the strong learner is then minimized based on the computed contribution.

As the dataset is limited, 5-fold cross-validation was used to assess the Gradient-Boosting model and test its performance. Applying the cross-validation approach, we divided the data into 5 subsets, where all subsets except one were used to train the model, while the one left was used for testing. This procedure was repeated 5 times, where at each iteration a different subset was left out for testing. A probability threshold of 50% was used for predictions. This means that if we consider malignant cases as 1s and benign cases as 0s, and the probability that the case was 1 was higher than 50%, the algorithm will classify it as malignant, whereas the case will be classified as benign if the probability that the case is 1 is lower than 50%.

Sensitivity, specificity and accuracy were calculated for the whole dataset, as well as for subgroups of interest that were previously mentioned. The sensitivity (true-positive rate) of the device is defined as the probability of the malignant cases being classified as malignant, while the specificity (true negative) refers to the probability that a benign case is classified as benign. Accuracy is defined based on how well the model can correctly classify both benign and malignant lesions.

**Figure 2:**
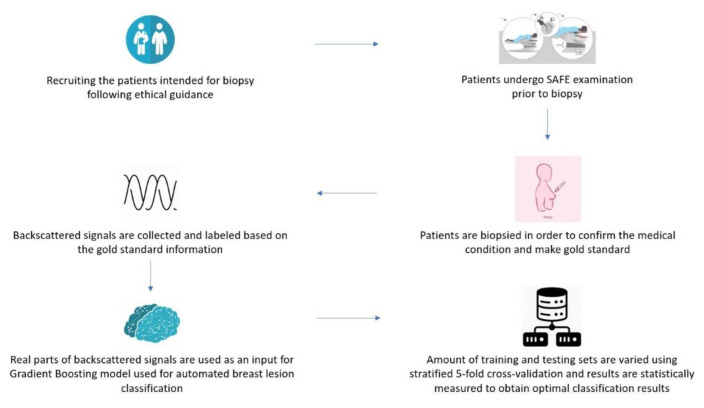
Flowchart of the proposed approach for MBI automated breast lesion classification.

A diagrammed flowchart of the complete SAFE clinical procedure is given in Figure 2.

## 3. Results

Fifty-four patients were included in the study. The patients’ ages ranged between 23 and 89 years. The average patient age was 44 years. The dataset contained 29 benign and 25 malignant lesions. Of the 29 benign findings, 10 were fibroepithelial lesions. In addition to fibroepithelial lesions, fibroadenoma was present in four cases; complex fibroadenoma, adenosis and granulomatous mastitis were present in two cases each; lacteal change, abscess, cellular fibroadenoma, apocrine metaplasia, focal chronic inflammation and necrosis had one case each. Three patients were characterized with normal findings. In malignant cases, invasive ductal carcinoma was the most dominant histopathological outcome, with 19 cases in total. Metastatic carcinoma, ductal carcinoma in situ, invasive lobular carcinoma and leiomyosarcoma were present in one case each. For two malignant cases, histopathology was unknown, as a biopsy was performed at another medical institution. Fifteen patients had non-dense breasts. The same number of patients were characterized by dense breast tissue. Breast density was unknown for 24 patients, as only ultrasound was available. A total of 21 patients were in the younger patient group, while 33 were older patients. A total of 32 patients had small breasts, while 22 of them were characterized as having larger ones. All dataset details are given in Table 1.

**Figure 3:**
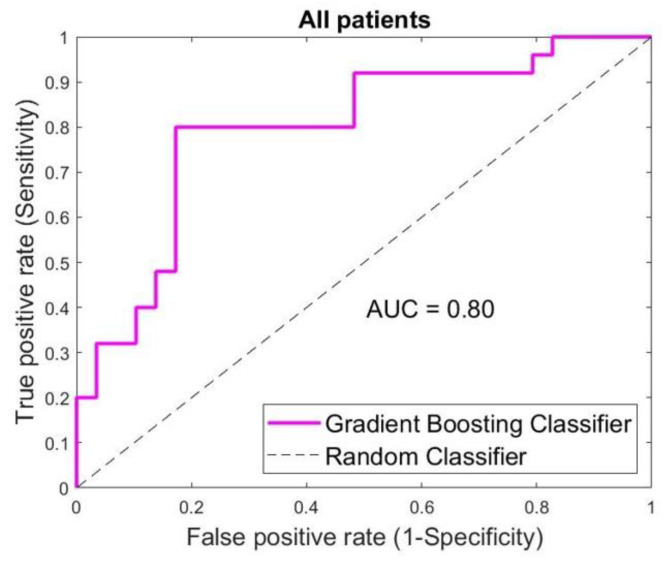
Classification outcome for all patients involved in the study. SAFE correctly classified 44 out of 54 lesions achieving sensitivity, specificity and accuracy of 80%, 83% and 81%, respectively.

Considering all patients involved, 44/54 lesions were classified correctly. From 10 cases which were not classified correctly, 5 of them were benign (false positive), and 5 were malignant (false negative). A total of 44 cases were classified correctly, comprising 20 malignant cases (true positive) and 24 benign cases (true negative). The sensitivity, specificity and accuracy achieved were 80%, 83% and 81%, respectively. The classification outcome was presented in Figure 3.

**Figure 4:**
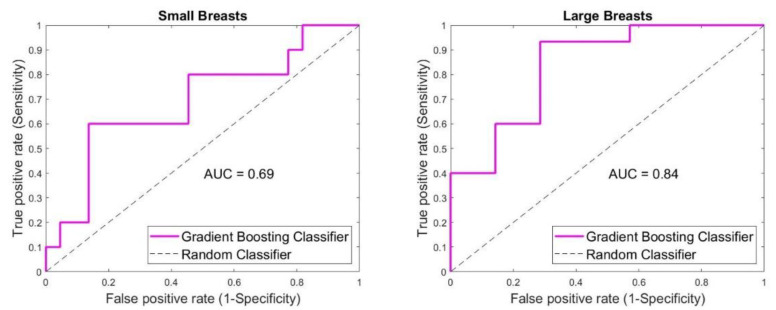
SAFE classification outcome based on the patient’s breast size. Device sensitivity and specificity were affected by the breast size, whereas lower sensitivity of 60% was noticed in smaller breasts compared to the 93% acquired in larger breasts. Contrary higher specificity of 86% was achieved in smaller breasts compared to the specificity of 71% in larger breasts. Higher accuracy was noticed in larger breasts (86% vs. 78%).

Figure 4 shows the effect of breast size on the classification outcome. In small breasts (cup sizes 1 and 2), 25/32 cases were classified correctly. Most of the correctly classified cases were benign ones, with 19 correctly classified cases (true negative) and only 3 falsely classified ones (false positive). Six of malignant cases were correctly classified (true positive), with four falsely classified ones (false negative). Low sensitivity of 60% was achieved. On the contrary, a high specificity of 86% was acquired. Accuracy for small breasts was 78%. When larger breasts (cup sizes 3 and 4) were involved, 19/22 cases were classified correctly. Contrary to small breast cases, most of the correctly classified ones were from the malignant group with 14 true-positive and 1 false-negative case. From the benign group, 5 true-negative and 2 false-positive cases were acquired. A high sensitivity of 93% with a lower specificity of 71% were achieved. Accuracy was 86%.

**Figure 5:**
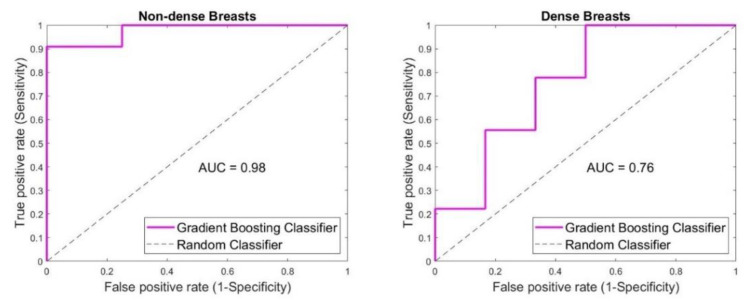
Density effect on SAFE classification outcome. Higher sensitivity, specificity and accuracy (91%, 100% and 93%, respectively) were achieved when non-dense breasts were involved compared to the ones acquired in dense breasts (56%, 83% and 67%, respectively).

SAFE performance considering the breast density was shown in Figure 5. In the case of non-dense breasts, 14/15 breast lesions were classified correctly, with 10 true-positive, 1 false-negative, 0 false-positive and 4 true-negative cases. Sensitivity of 91% and accuracy of 93% were achieved, while specificity was 100%. In the case of dense breasts, 10/15 breast lesions were classified correctly, with 5 true-positive, 5 true-negative, 1 false-positive and 4 false-negative cases. Accuracy, sensitivity and specificity of 67%, 56% and 83% were achieved, respectively.

**Figure 6:**
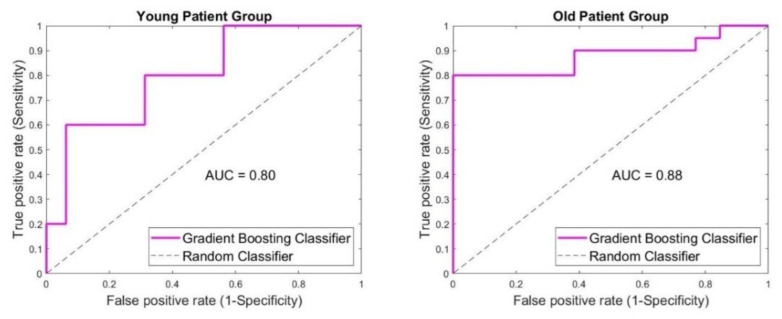
Representation of SAFE performance according to the patient’s age. Considering the younger group, sensitivity, specificity and accuracy achieved were 80%, 69% and 71%, respectively. Higher percentages were noticed in older group, where the sensitivity, specificity and accuracy achieved were 80%, 100% and 88%, respectively.

The analysis regarding the effect of the patient’s age on the classification outcome is shown in Figure 6. Considering younger patients, 15/21 cases were classified correctly. Most of the correctly classified cases were benign (11 true-negative and 5 false-positive cases). When malignant lesions were involved, only 4 cases were classified correctly (true positive) while 1 case was falsely classified (false negative). Sensitivity of 80% was achieved, while specificity was 69%. The acquired accuracy was 71%. When older patients were involved, 29/33 cases were classified correctly. A total of 16 malignant (true positive) and 13 benign (true negative) cases were classified correctly. Four malignant (false negative) and none of the benign (false positive) cases were falsely classified. Accuracy, sensitivity and specificity were 88%, 80% and 100%, respectively.

**Figure 7:**
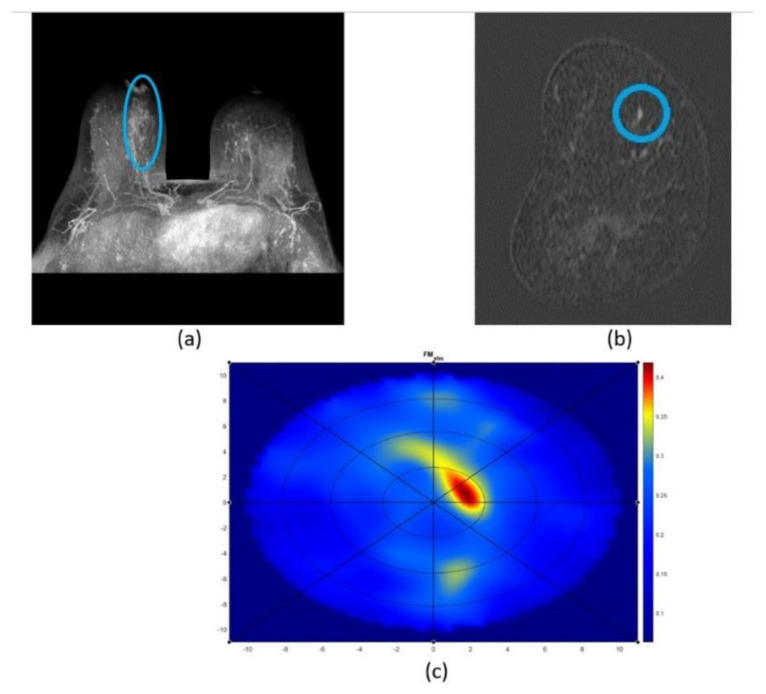
Imaging performance of MRI and SAFE: (**a**,**b**) MRI axial and coronal planes showing the presence of malignant lesion in non-dense breast, and (**c**) SAFE coronal plane confirming the presence of malignant lesion (high contrast region), inside the scanning domain (blue circle), at the same location as reported by MRI.

**Figure 8:**
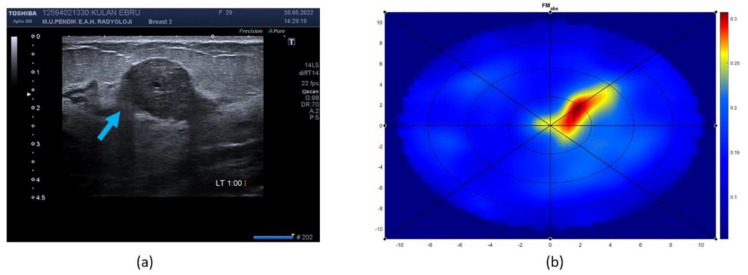
Imaging performance of ultrasound and SAFE: (**a**) ultrasound scan axial showing the presence of benign lesion in small breast of unknown density, and (**b**) SAFE scan confirming the presence of benign lesion (high contrast region), inside the scanning domain (blue circle), at the same location as reported by ultrasound.

Example cases of MBI imaging outcomes compared to conventional imaging are represented in Figure 7 and Figure 8. Figure 7 represents an older patient, who was diagnosed with a malignant lesion (invasive ductal carcinoma). The breast was characterized as a non-dense and large one. SAFE confirmed the biopsy outcome and classified the present lesion as a malignant one. Additionally, SAFE and MRI confirmed the presence of the lesion at the same location. With SAFE, lesions are characterized by the high-contrast (red) region. Figure 8 represents a younger patient, who was diagnosed with a benign lesion (fibroadenoma). The breast was characterized as a small one with unknown density information, as the patient was examined with ultrasound. As in the previous case, SAFE confirmed the biopsy outcome, classifying the lesion as benign. In addition, the location reported with ultrasound was confirmed by SAFE.

## 4. Discussion

The MBI screening device was developed for automated breast lesion classification. Compared with the previous version of our device [35], a new VNA was employed to provide better portability. Additionally, a broader frequency range was (0.6–8 GHz) employed, and the time of the scan was cut to half of the previous prototype’s time (from 20 to 10 min per patient). Besides the fact that more scans can be performed per day, shorter scanning times decrease the chance of patient movement during the examination. As movement can be a cause of increased noise in the data, shorter scanning can lead to less noisy datasets.

A machine learning model, namely Gradient Boosting, was trained using the real part of the *S*_21_ matrix, which represents the raw data acquired from the scanning. Results show that the new version of our MBI device, namely SAFE, when augmented with the ML approach, is capable of classifying anomalies present in the breasts of patients with an accuracy of 81%, sensitivity of 80% and specificity of 83%. These percentages represent an indication that SAFE can be used to provide the breast tissue pathology of patients of any age without safety restrictions due to its non-invasive and non-ionizing nature. This study involved 54 patients, from which 29 had benign and 25 had malignant findings. Density had a high impact on the classification results, where higher percentages were noticed in non-dense breasts. Sensitivity and accuracy were considerably affected by the density of the breast, where sensitivity and accuracy of 56% and 67% were achieved in dense breasts, respectively. On the contrary, sensitivity and specificity were considerably higher in non-dense breasts, where the ML model acquired values of 91% and 93%, respectively. These results are to be expected, as the dielectric contrast between fat (more dominant in non-dense breasts) and breast anomaly is considerably higher than the one between the glandular tissue (more dominant in dense breasts) and present anomaly. The sensitivity of the device was also considerably affected by the breast size, where in smaller breasts the overall sensitivity achieved was 60%, compared to 93% in larger ones. Better results in larger breasts may be connected with the electromagnetic interaction between the breast and the antennas. In cases of smaller breasts, it is possible that direct coupling between antennas may increase, making it very hard to differentiate small variations of the fields, which may affect the device's capability to correctly classify malignant lesions. Patient age had a considerable effect on the specificity and accuracy of the MBI device. While the high specificity of 100% and accuracy of 88% were achieved in the older patient group, lower specificity (69%) and accuracy (71%) were noticed in the younger patient group. It is well known that the density of breasts decreases with age, which can explain the correlation between the results of the age and density subgroups.

Considering the main subgroups of interest, such as the young patient group and the dense breast group, in the areas where mammography—as the gold standard for breast screening—is limited, we posit that the Gradient-Boosting algorithm may not be appropriate for MBI automated breast lesion classification. This may be the case even though the overall sensitivity, specificity and accuracy rates were high. Additionally, with the Gradient-Boosting algorithm, higher numbers of false-negative cases compared to false-positive ones were noticed, which can lead to a false sense of security that can cause doctors to believe the patient does not need treatment. However, as Gradient Boosting was performed on a small dataset, the question of whether the limited dataset for the subgroups of interest affects the model’s overall performance should be further investigated. The issue regarding a higher number of false-negative cases compared to false-positive ones, in most of the subgroups analyzed, will be addressed by modifying the Gradient-Boosting model and performing advanced research on feature selection. The ML study will continue as a part of a current clinical trial, where additional data collected will be used to enhance the proposed model and classification outcome.

## Figures and Tables

**Table 1:** Details of the dataset analyzed in the study.

Patient Group	Number of Cases
All cases	54
Benign cases	29
Malignant cases	24
Small breast cases	32
Large breast cases	22
Non-dense cases	15
Dense cases	15
Young cases	21
Older cases	31

## Data Availability

The data are not publicly available due to Governmental and Institutional (Marmara University Hospital Breast Center) regulations and non-violation of patient’s privacy.
